# Surveillance and simulation of bovine spongiform encephalopathy and scrapie in small ruminants in Switzerland

**DOI:** 10.1186/1746-6148-6-20

**Published:** 2010-04-18

**Authors:** Chantal Häusermann, Heinzpeter Schwermer, Anna Oevermann, Alice Nentwig, Andreas Zurbriggen, Dagmar Heim, Torsten Seuberlich

**Affiliations:** 1 NeuroCenter, Reference Laboratory for TSE in animals, Department of Clinical Research and Veterinary Public Health, Vetsuisse Faculty, University of Berne, Switzerland; 2 Federal Veterinary Office, Liebefeld, Switzerland

## Abstract

**Background:**

After bovine spongiform encephalopathy (BSE) emerged in European cattle livestock in 1986 a fundamental question was whether the agent established also in the small ruminants' population. In Switzerland transmissible spongiform encephalopathies (TSEs) in small ruminants have been monitored since 1990. While in the most recent TSE cases a BSE infection could be excluded, for historical cases techniques to discriminate scrapie from BSE had not been available at the time of diagnosis and thus their status remained unclear. We herein applied state-of-the-art techniques to retrospectively classify these animals and to re-analyze the affected flocks for secondary cases. These results were the basis for models, simulating the course of TSEs over a period of 70 years. The aim was to come to a statistically based overall assessment of the TSE situation in the domestic small ruminant population in Switzerland.

**Results:**

In sum 16 TSE cases were identified in small ruminants in Switzerland since 1981, of which eight were atypical and six were classical scrapie. In two animals retrospective analysis did not allow any further classification due to the lack of appropriate tissue samples. We found no evidence for an infection with the BSE agent in the cases under investigation. In none of the affected flocks, secondary cases were identified. A Bayesian prevalence calculation resulted in most likely estimates of one case of BSE, five cases of classical scrapie and 21 cases of atypical scrapie per 100'000 small ruminants. According to our models none of the TSEs is considered to cause a broader epidemic in Switzerland. In a closed population, they are rather expected to fade out in the next decades or, in case of a sporadic origin, may remain at a very low level.

**Conclusions:**

In summary, these data indicate that despite a significant epidemic of BSE in cattle, there is no evidence that BSE established in the small ruminant population in Switzerland. Classical and atypical scrapie both occur at a very low level and are not expected to escalate into an epidemic. In this situation the extent of TSE surveillance in small ruminants requires reevaluation based on cost-benefit analysis.

## Background

Transmissible spongiform encephalopathies, in sheep and goats (small ruminants) include classical and atypical scrapie as well as BSE [1]. Common denominators of TSEs are the lethal neurodegenerative alteration of the CNS that involves spongiform lesions, neuronal loss, gliosis and the accumulation of a conformational abnormal isoform (PrP^d^) of the physiological prion protein (PrP^c^) [2]. The classical type of scrapie has been a threat in the European sheep livestock for centuries, its first records dating back to the 18^th ^century [3]. Classical scrapie is a contagious disease, infectivity was detected in the CNS, lymphatic tissues, several organs and body fluids of affected animals and its transmission occurs vertically as well as horizontally within and between flocks [4-6]. By contrast atypical scrapie that was first identified in Norway in 1998 (and is thus termed alternatively Nor98 scrapie, [7]) differs from classical scrapie in the type and distribution of neuropathological lesions and the biochemical characteristics of PrP^d ^from classical scrapie. PrP^d ^and infectivity have not yet been demonstrated outside the CNS in such affected animals and the disease does not appear to be contagious. This and epidemiological data [8-11] support the notion that atypical scrapie might have a spontaneous origin, similar to sporadic TSEs in humans [12].

BSE primarily affects cattle and the agent was recycled in the population by means of feeding supplements that contained contaminated meat and bone meal (MBM) [13]. Since 1996, BSE is regarded as a zoonotic disease [14-17].

Sheep and goats were also exposed to BSE contaminated MBM during the BSE epidemic and proved to be susceptible after experimental oral exposure [18]. In contrast to the situation in cattle, the agent also transmitted vertically in an experimental setup [19]. In 2005 the first natural case of BSE was reported in a goat in France [20] and despite a number of suspicious cases [21] this remained the only confirmed case for the time being. As clinical signs and histopathologic lesions in BSE affected sheep and goats are indistinguishable from scrapie, concerns were raised that BSE established in these populations unrecognized, presenting a risk for consumers and undermining disease control measures.

Switzerland was one of the first countries to recognize a BSE epidemic in the cattle population outside the United Kingdom in 1990 [22]. This epidemic peaked in 1995 and faded out in 2006 after a total of 465 detected cases. In 1990 the feeding of MBM was banned for all ruminants and constantly reinforced until the complete ban of MBM in all farm animal feed in 2001.

Before 1990 a single case of TSE, at that time classified as scrapie, had been diagnosed in Switzerland in a goat in 1981([23], table 1), and thus little was known about its prevalence in this population. Because of the putative etiological link between scrapie and the BSE epidemic and to estimate the prevalence more precisely, TSEs in small ruminants in Switzerland were subject of passive surveillance since 1990 and active surveillance since 1998. By these means in total 16 cases of small ruminant TSE had been identified (table 1). A proportion of them dated from the early 1990s when laboratory procedures to distinguish between scrapie and BSE had not yet been available and atypical scrapie was unknown. It was thus still unclear whether either type of scrapie or rather BSE affected these animals.

**Table 1:** Surveillance for TSE in small ruminants in Switzerland, 1990-2008.

Year	Passive surveillance		Active surveillance		
	Analysis	TSE cases	Analysis		TSE cases
			Fallen stock	Slaughter	
1981-1990	-	1	-	-	-
1991	25	1	-	-	-
1992	21	-	-	-	-
1993	15	3	-	-	-
1994	40	-	-	-	-
1995	73	1	-	-	-
1996	93	-	-	-	-
1997	38	-	-	-	-
1998	68	-	77^1^	-	-
1999	85	1	381^1^	-	-
2000	91	-	407^1^+ 71^2^	-	-
2001	66	-	255^2^	-	-
2002	104	-	4^2^	49^3^	-
2003	36	-	-	404^3^	-
2004	80	1	1807^5^	18004^4^	6
2005	23	-	1386^5^	14773^4^	2
2006	7	-	-	-	-
2007	11	-	-	-	-
2008	10	-	-	-	-

Total	886	8	4388	33230	8

Annual numbers of animals analyzed in passive and active surveillance as well as numbers of confirmed TSE cases are indicated.

^1^tested with Prionics Check Western

^2^tested with Prionics Check Western and IHC and histopathology in parallel [37]

^3^tested with Bio-Rad TeSeE sheep and goat ELISA IHC and histopathology in parallel [36]

^4^tested with Bio-Rad TeSeE sheep and goat ELISA or Prionics Check Western SR [28]

^5^tested with Bio-Rad TeSeE sheep and goat ELISA, Prionics Check Western SR, IHC and histopathology in parallel [28]

By legislation, control measures are implemented on flock level, whenever index cases are detected. In most of the outbreaks since 1990 flock mates were culled and analyzed by at that time state-of-the-art laboratory diagnostic procedures. No secondary cases were identified in these flocks. However, knowledge on TSEs increased over time and more sensitive diagnostic tools were developed. It is possible, that especially in the 1990s, secondary cases had been overlooked.

Herein we present a comprehensive compilation of the TSE surveillance activities in small ruminants in Switzerland since 1990. By using timely diagnostic procedures we reanalyzed the status of the complete set of historic small ruminant TSE cases and most of their flock mates. Based on these data we then calculated the prevalence distributions for BSE, classical and atypical scrapie. To combine the evidence of the absence of BSE and low scrapie prevalence, that has been collected by different surveillance systems over a long period of time, a stochastic, flock explicit model was developed to predict prevalence changes in the long run over the next 70 years. The aim of this study was to come to an overall assessment of the current small ruminant TSE situation in Switzerland and to forecast its future development.

## Methods

### Tissue samples

Paraffin-wax embedded CNS tissues of all small ruminant TSE cases which were identified between 1981 and 1999 as well as a number of their flock mates were available from the archives of the NeuroCenter, Vetsuisse Faculty, University of Berne and the Institute of Veterinary Pathology, Vetsuisse Faculty, University of Zürich (kindly provided by F. Ehrensperger and M. Hilbe). For case 30842 and the flock mates of goat 22614 we had access to brain tissue homogenates. Sheep S7/CS as well as the TSE cases which were detected in the frame of the active TSE surveillance program in 2004 and 2005 were described previously [24,25]. However, the analysis of the flock mates of these animals is reported in the present study. For these animals frozen and paraffin-embedded tissues of the brain, the retropharyngeal lymph nodes and the tonsils were available.

Classical scrapie confirmed obex wax blocks derived from two clinically affected sheep, one of the ARQ/VRQ, the other of unknown PrP genotype and were kindly provided by M. Bardsley (TSE Archive, VLA Weybridge, UK). Ovine BSE control tissue was brainstem from a clinically affected Suffolk sheep of the ARQ/ARQ genotype (BSE^ov^-1) intracerebrally (*i.c*.) inoculated with first passage ovine BSE and kindly provided by P. Berthon and F. Lantier from INRA Tours-Nouzilly, France. Spinal cord from two bovine BSE *i.c*. challenged sheep of the ARQ/ARQ genotype (BSE^ov^-2 and BSE^ov^-3), were kindly provided by M.M. Simmons (VLA Weybridge, UK).

### Immunohistochemistry

The immunohistochemical method (for details see additional file 1) was performed as described by Jeffrey et al [26] based on PrP^d ^epitope mapping allowing for discrimination of classical scrapie and small ruminant BSE. To this end, two monoclonal antibodies with affinity to different epitopes were selected: N-terminal mouse MAb 12B2 (_93_WGQGG_97_, 0,23 μg/ml, kindly provided by Jan P.M. Langeveld, CVI Wageningen, the Netherlands) and C-terminal rat MAb R145 (_222_RESQA_226_, 2 μg/ml, VLA, UK). As a result of distinct intracellular truncation of PrP^d ^in BSE and classical scrapie, characteristic PrP labeling appears: in case of classical scrapie intracellular labeling is visible with both antibodies whereas small ruminant BSE is characterized by intracellular staining only with MAb12B2. Further analyses of unconfirmed cases were accomplished by using a panel of the following MAbs: i. P4 (0,125 μg/ml, R-Biopharm AG), ii. F89/160.1.5 (1 μg/ml, VMRD), iii. L42 (1 μg/ml, R-Biopharm), iv. SAF84 (0,5 μg/ml, SPI-bio) and v. F99/97.6.1(1 μg/ml, VMRD). For the analysis of flock mates of the TSE cases we applied either MAb 2G11 (1,25 μg/ml, Institut Pourquier) or F99/97.6.1.

### Discriminatory Western Blot (WB)

The discriminatory WB was carried out on the basis of a commercial BSE rapid test (Check Western, Prionics) and in principle as described previously by M. Stack and colleagues [27]. The selected MAbs were the PrP core-binding 6H4 (0,2 μg/ml, Prionics) and the N-terminal-binding MAb P4 (0,1 μg/ml).

### TSE screening tests

Two commercial TSE screening tests, the Bio-Rad TeSeE sheep and goat ELISA (Bio-Rad, France) and the Prionics Check Western SR (Prionics, Switzerland), were applied to brain tissue homogenates according to the manufacturer's instructions. Both tests were evaluated and approved for the purpose of TSE surveillance in small ruminants using obex tissue samples by the Swiss Federal Veterinary Office.

It must be stated that the protocol of the Prionics Check Western SR that had been applied in 2004 and 2005 in the Swiss active surveillance differed from that of a test of the same name approved in the EU since 2006 in a shorter incubation time and temperature during the PK digestion (37°C and 30 min instead of 48°C and 60 min). The field performances of the Bio-Rad TeSeE sheep and goat ELISA and the Prionics Check Western SR in the 2004-2005 active surveillance program have been described recently [28].

### Genotyping

The PRNP genotypes of the flock mates of some cases were determined by direct DNA sequencing by a commercial service (Medigenomix, Martinsried, Germany).

### Prevalence calculation

To estimate the situation for BSE, classical scrapie and atypical scrapie after the survey in 2004/2005 we conducted a Bayesian prevalence calculation, where the informed prior was given by a pert distribution. The parameters for the pert distribution were derived from the negative results of testing 859 sheep from fallen stock in the years 1999 and 2000 (table 1). To exclude unrealistic high numbers of infected animals from the prior distribution, the upper values of the prior distribution were set to the maximum values of 50 infected animals for BSE and 1'000 infected animals for both types of scrapie. We estimate these values as highly conservative, as these values correspond to prevalences of 356/100'000 for scrapie and 18/100'000 for BSE, respectively. For classical scrapie, this upper limit would equal the situation in UK before 2003 [29], and is higher than the highest observed prevalence for atypical scrapie in the EU [11]. For BSE, the prior value is equal to the highest estimated annual BSE prevalence in cattle in Switzerland in the year 1995 [30]. Thus the prior distributions were Pert (0,0,0.000178) for BSE and Pert (0,0.000006,0.00356) for classical scrapie and atypical scrapie. The calculation of the posterior prevalence distribution follows a binomial function [31].

### Simulation model

We built simulation models for BSE, classical scrapie, and atypical scrapie separately. The models were identical, except for the prevalence distribution used for the number of infected animals in the first year. The period covered was 70 years, with a calendar year as single time step. We assumed a constant population (flocks, animals) over time. For each model, the mean probability, 5^th ^and 95^th ^percentiles were derived from 10'000 iterations. The space between the 5^th ^and the 95^th ^percentile is equal to 90% of the values of each.

The unit of interest in the model is the flock. However on each flock, numbers of infected and uninfected small ruminants are simulated. There are two key processes related to each flock in the model. First, the probability for an animal in an uninfected flock of acquiring infection (*λ*_*j*_) is solely dependent on the movement of infected animals between flocks, given by:(1)

where *i *and *I *are the number of infected flocks and total flocks, *a*_*i *_is the number of infected animals in the population, *n*_*i *_is the total number of animals in infected flocks and ϕ is the probability of transmission between flocks given an infected animal moved from an infected to the uninfected flock. Thus, the number of new infected small ruminants (sr_newinf_) in TSE-free flocks follows the binominal function:(2)

The second important process, generating the number of secondary infected small ruminants sr_secinf_, is calculated for each flock by:(3)

where *i*_*j *_is the number of infected small ruminants in the flock, *R*_0 _is the basic reproduction number for TSE, *G *is the incubation period and *s *is the flock level susceptibility. The latter variable incorporates the variation of susceptibility due to differences in the genotype distributions on farm level into the model and was derived from UK sheep farms prior to the genotype breeding program [32]. There is no indication for anomalous composition of the genotypes in the sheep population in Switzerland ([33], and see additional file 2). Thus it is unlikely that the flock level susceptibility is ill-parameterized. As sr_secinf _can exceed the flock size, an artificial upper boundary was included in the model, assuming that flocks with more than 50% of the animals infected would be detected, culled, and restocked with negative animals. For each infected animal, a fixed pair of *R*_0 _and *G *was determined. As we assumed no change in the proportions of scrapie related genotypes over time, because no program for breeding towards scrapie resistance was in place in Switzerland, the flock level susceptibility *s *was determined once for each flock per iteration. In all models, we did not include the detection probability of cases, which depends on the incubation period, the sample quality and the diagnostic characteristics of the tests used. To derive the number of infected animals, all infected animals in the respective year are summed up, either in a single flock or the whole population. The number of infected flocks is given be the sum of flocks were at least one infected animal is present in the time-step.

### Sensitivity analysis

The parameters for the variables were derived from a model for classical scrapie in GB [32]. As these parameters have been estimated for classical scrapie only, we used the same parameters for atypical scrapie and BSE. We conducted a sensitivity analysis to study the influence of variables determining the between flock transmission (ϕ) in contrast with variables determining the within flock transmission (R0, G, s), and the population structure. In table 2, the original used parameters for ϕ, R0, G and s as well as the parameters used in the sensitivity analysis are given. In the sensitivity analysis, the seed was fixed at 100 infected animals.

**Table 2:** Parameters of variables used in the simulation models for small ruminant BSE, atypical scrapie and classical scrapie and in the sensitivity analysis.

Model	Variable	Parameter
BSE, atypical and classical scrapie according to [32]	seed	min- 95^th ^% percentile from prevalence calculation
	ϕ	uniform (0.0002,0.014)
	R0	uniform (2.5,14)
	G	uniform (1.6,5.9)
	s	uniform (0.002,0.14)

High between flock transmission	Seed	100
	ϕ	0.03
	R0, G, s	unaltered

High within flock transmission	seed	100
	ϕ, G, s	unaltered
	R0	uniform(12,14)

Low within flock transmission	seed	100
	ϕ, G, s	unaltered
	R0	uniform(2.5,5)

### Data and software

The population data were extracted from the agricultural census 2004 (Swiss Federal Statistical Office). The simulation models and the prevalence calculation were implemented in Microsoft Excel (^© ^Microsoft) together with an add-in package @Risk (^© ^Palisade Corp.) version 4.5.2.

## Results

### First case of classical scrapie in 1981

**Figure 1:**
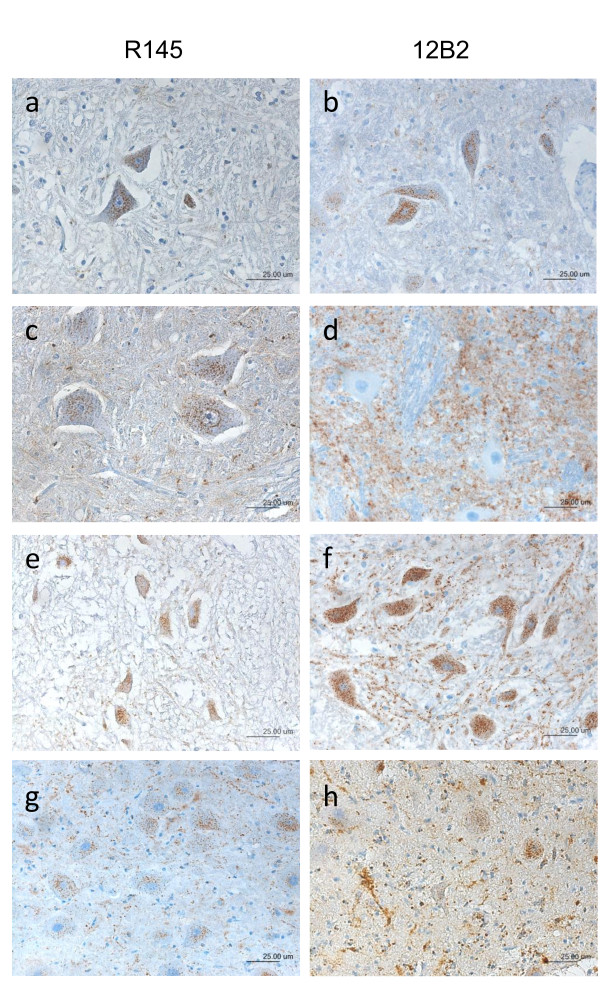
**Discriminatory PrP^d ^immunohistochemistry**. Classical Scrapie (a+b) and ovine BSE (c+d) are discriminated by using two antibodies linking at distinct epitopes of the prion protein. The core-binding MAb R145 shows intraneuronal immunolabeling in classical scrapie (a) and ovine BSE (c) while the n-terminal-binding MAb 12B2 only detects intraneuronal labeling in classical scrapie (b) whereas PrP^d ^in ovine BSE remains undetected in the neurons. Two examples, one of a goat (ID 15355, e and f) and one of a sheep (ID S1RS, g and h) exemplify the findings in the cases under investigation. For the results of the complete sample set refer to table 4.

The first case of small ruminant TSE in Switzerland was suspected in a goat in 1981 ([23], table 3). This animal showed progressive scrapie associated neurological signs. The flock of origin was monitored as part of a research project on caprine arthritis-encephalitis (CAE). The brain of this goat was subjected to a neuropathologic examination and revealed spongiform lesions in the basal ganglia, thalamus, midbrain and the nuclei of the medulla oblongata leading to the diagnosis of scrapie in this animal. For the present study we were able to retrieve paraffin embedded obex brain tissue of this goat from our archives and analyzed it for the presence of PrP^d ^by discriminatory IHC. Intraneuronal immunolabeling was evident with both MAbs similar to the classical scrapie control, but clearly different from the ovine BSE controls, in which MAb 12B2 shows no intraneuronal labeling (figure 1, table 4). Regardless of the antibody applied, intraneuronal labeling has never been observed in atypical scrapie cases [34,35] and therefore indeed this animal was retrospectively confirmed as the first case of classical scrapie identified in Switzerland.

**Table 3:** Complete set of historical TSE cases in small ruminants identified in Switzerland.

Surveillance stream	Case ID	Year of Diagnosis	Species	Age (years)	Sex	PRNP Genotype	Primary diagnosis	Tested flock mates
-	15355	1981	goat	3	f	nd	scrapie^1^	-

passive	21658	1991	sheep	4	f	nd	scrapie^1^	18
	22614	1993	goat	2	f	nd	scrapie^1^	25
	22805	1993	sheep	> 1.5	nr	nd	scrapie^1^	17
	22856	1993	sheep	6	f	nd	scrapie^1^	3
	24058	1995	sheep	1.5	f	nd	scrapie^1^	11
	30842	1999	sheep	3.5	m	VRQ/VRQ	scrapie^2^	19
	S7/CS	2004	sheep	6	f	ARQ/ARQ	atypical scrapie^3^	31

active	G1/RS	2004	goat	10	f	AHQ/AHQ	atypical scrapie^3^	17
	G2/FS	2004	goat	12	m	AHQ/AHQ	atypical scrapie^3^	1
	S1/RS	2004	sheep	nr	f	VRQ/VRQ	classical scrapie^3^	42
	S2/RS	2004	sheep	nr	f	ARR/ARR	atypical scrapie^3^	169
	S3/RS	2004	sheep	nr	f	ARR/ARR	atypical scrapie^3^	3
	S4/RS	2004	sheep	10	f	AHQ/AHQ	atypical scrapie^3^	-
	S5/FS	2004	sheep	6	f	AHQ/ARQ	atypical scrapie^3^	30
	S6/FS	2005	sheep	> 1.5	f	ARR/ARR	atypical scrapie^3^	16

The primary diagnosis was established by state-of-the-art techniques at the year of confirmation and for cases identified before 2004 involved no means to discriminate BSE and atypical scrapie from classical scrapie.

^1^confimed by histopathology

^2^confimed by histopathology and immunohistochemistry

^3^confirmed by immunohistochemistry and subjected to biochemical TSE typing by Western immunoblot in a previous study [24].

G, goat; S, sheep; RS, regularly slaughtered; FS, fallen stock; CS, clinical suspect; nd, not done; nr, not reported; f, female; m, male

**Table 4:** Results of the discriminatory immunohistochemistry in historical TSE cases from passive surveillance.

Samples	ID	CNS region	Intraneuronal PrP^d ^labeling	
			MAb R145	MAb12B2
Controls	Classical scrapie	Obex	+++	+++
	BSE^ov ^- 1	Obex	+++	-
	BSE^ov ^- 2	Spinal cord	+++	-
	BSE^ov ^- 3	Spinal cord	+++	-
	TSE-negative	Obex	-	-

Pre-surveillance	15355	Obex	+++	+++

Passive surveillance	21658	Hippocampus	+	+++
	22614	Midbrain	+	++ - +++
	22805	Midbrain, hippocampus	+	++
	22856	Obex	-	-
	24058	Cerebral cortex	-	-
	30842	Spinal cord	+ - ++	+++

### Passive TSE surveillance

**Figure 2:**
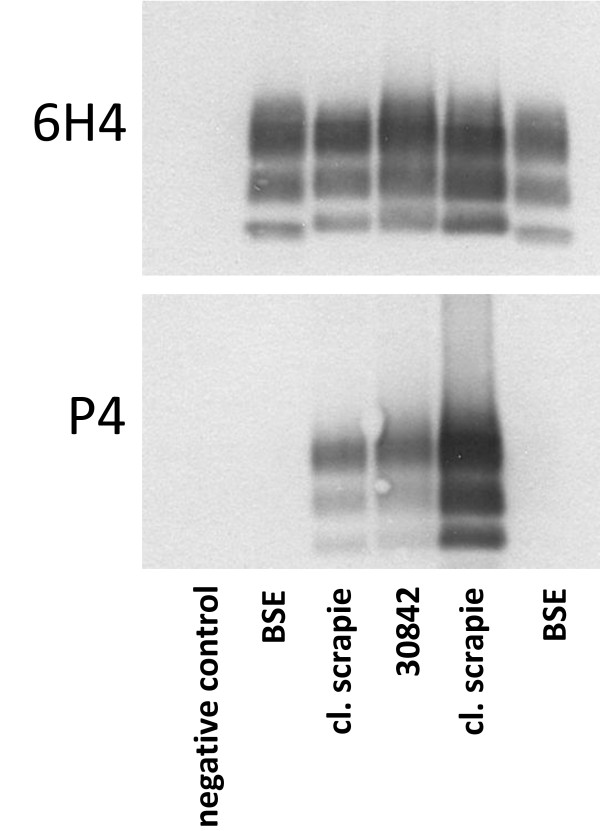
**Discriminatory Western Immunoblot for case 30842**. Brain tissue homogenate of sheep 30842 along with BSE-positive, scrapie-positive and negative controls was digested with proteinase K and MAbs P4 and 6H4 were used for PrP^res ^detection and discrimination of BSE and scrapie by Western immunoblot. The pattern of the di-, mono- and non- glycosylated moieties of case 30842 shows typical features of classical scrapie with regard to molecular mass of non-glycoslated PrP^res ^(lower band) and the reactivity with the n-terminal MAb P4.

As shown in table 1, the number of reported clinically TSE suspicious small ruminants increased between the implementation of the passive surveillance in 1990 and 2004 and then decreased to levels of some 10 animals per year in the last three years. Out of a total of 886 animals investigated, six sheep and one goat were confirmed with a TSE (at that time designated scrapie, table 3). The clinical signs in these animals ranged from apathy over paresis of the limbs, tremor, itching, nibbling response, and anxiety to recumbency and breaking down. While in each case the initial confirmatory laboratory procedure was based on the histopathologic examination of brain sections, for the most recent case (S7/CS) further investigations using IHC and WB found this animal to be affected by atypical scrapie [24]. To determine whether any of the remaining six cases was affected by BSE, we analyzed archived paraffin embedded brain tissue blocks by the discriminatory IHC. Intraneuronal PrP^d ^deposits were readily identified in all but two cases with MAb 12B2 and MAb R145 (figure 1 and table 4), again indicating that these animals were affected by classical scrapie. For case 30842 frozen brainstem tissue was available and subjected to a WB procedure that, based on different N-terminal PK cleavage sites in PrP^d^, allows for discriminating ovine BSE from classical scrapie. In agreement with the IHC results, this animal was scored as classical scrapie (figure 2). In the two cases where the discriminatory IHC was negative with both MAbs (case 24058 and 24856), we extended the IHC analysis with a panel of additional PrP specific MAbs to tissue blocks of all CNS structures available. This comprised the obex, cerebellum and cerebral cortex for sheep 24856, but was limited to cerebral cortex in sheep 24058. In none of the samples PrP^d ^was detected and therefore these two cases remained unconfirmed and could not be further characterized.

### Active TSE surveillance

In several pilot studies conducted on fallen stock and slaughtered small ruminants between 1998 and the end of 2003 no TSE cases were found (table 1). However, TSE-unrelated histopathologic changes indicative for other neurological disorders were found in about 15% of the fallen stock. By contrast, in healthy slaughtered small ruminants this proportion was significantly lower with 5% [36,37]. These findings point out the importance of the fallen stock as target population for the surveillance of neurological diseases in small ruminants and served to design a large one-year active surveillance program that had subsequently been enforced between June 2004 and July 2005. Special emphasis was placed on in-depth examination of adult fallen animals. Over 3'000 fallen sheep and goats, which represent approximately 1% of the total adult (> 1 year of age) small ruminant population, were analyzed by two approved TSE screening tests in parallel on obex tissue samples and additionally by IHC on seven different brain levels and on retropharyngeal lymph nodes and tonsils. In addition, obex tissue samples of approximately 33'000 routinely slaughtered adult sheep and goats were analyzed using one of the two screening tests (for details see [28]). We described previously that this active surveillance program led to identification of 8 TSE cases, among which 7 were of atypical scrapie nature and a single one showed the characteristics of classical scrapie ([24], table 3).

### Culled flock mates

Similar to clinically suspicious animals, from the beginning of passive surveillance until 1998, the diagnosis in culled flock mates relied on histopathologic examination of the brain only. We now retrospectively analyzed from a total of 93 flock mates of the scrapie cases identified between 1990 and 1999 tissue blocks from brainstem, cerebellum, cerebrum, hippocampus, and midbrain by a timely IHC procedure. For the flock mates of a goat (ID 22614) only frozen samples of medulla oblongata, cerebellum, thalamus and basal ganglia were available. These were tested in the Bio-Rad TeSeE sheep and goat ELISA for the presence of PrP^d^. Flock mates of TSE cases identified after 1999, in total 309 animals, were analyzed more comprehensively. All were assayed by IHC in multiple brain levels and lymphoid tissues and TSE screening tests on obex samples. Due to logistical restrictions, for flock mates of S1/RS only obex tissues were subjected to the Prionics Check Western SR. In none of these herds secondary cases were identified.

### Bayesian prevalence calculation

The most likely estimates for the number of cases in the adult population after the one-year comprehensive survey in 2004/2005 were 2 for BSE (prevalence 1/100'000), 13 for classical scrapie (prevalence 5/100'000) and 59 for atypical scrapie (prevalence 21/100'000). The 95% confidence intervals were 0-15 cases for BSE, 1-41 cases for classical scrapie, 25-111 cases for atypical scrapie.

### Simulation model

**Figure 3:**
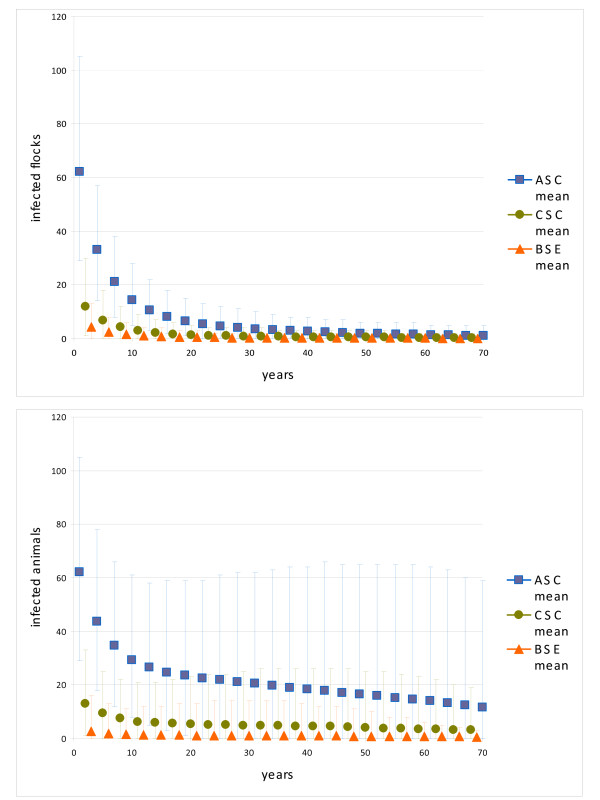
**Forecasting TSE prevalences in Switzerland**. Means (dots) and 90% probability intervals (light lines) of the number of infected animals and flocks forecasted over 70 years by the simulation models are shown for small ruminant BSE, classical scrapie (CSC) and atypical scrapie (ASC).

In all three models the mean number of infected animals dropped by half in the first 10 years but remained nearly stable thereafter (figure 3). After a sharp decrease, the 95th percentile values for the number of infected flocks decreased slightly, from 5 to 1 for BSE, 9 to 2 for classical scrapie and 26 to 6 for atypical scrapie from years 10 to 60. The prominent decrease in the first years is an artifact related to the seeding process, where the infection is randomly distributed in single flocks. If very small flocks are infected, the disease dies out rapidly in these flocks. Thus we focus our observation on the time steps after this initial phase. In contrast to atypical scrapie, for BSE and classical scrapie, no new flocks were infected at the 95th percentile after 7 and 27 years, respectively.

In the sensitivity analysis, the model with high between flock transmission behaved like the original model, the model with high within flock transmission led to an epidemic curve with a maximum in year 28, and in the model with low within flock transmission the disease died out after 39 years (all values for the 95^th ^percentiles). In single iterations we observed that the prevalence of disease was highly related to the likelihood of entering one of the few larger flocks. Therefore, we also run models with a hypothetical high number of larger flocks (> 100 animals). In these models, the disease was more likely to persist and produced larger epidemics, even with the model of low within flock transmission.

## Discussion

In the past 30 years 16 cases of TSE have been identified in Swiss sheep and goats. Seven cases date back to the 1990s, the decade of the highest BSE incidence in domestic cattle; it was not clear whether they were scrapie cases as assumed or rather the result of a BSE infection. Here we show now that in five of these animals the IHC labeling pattern was consistent with classical scrapie. Still in two sheep a TSE could not be confirmed by IHC. However, a definite evaluation of both cases is not feasible because either diagnostic target sites, obex for classical scrapie and BSE and cerebellar cortex for atypical scrapie, are missing or frozen unfixed material allowing proceeding to alternative tests such as ELISA or WB, is not available.

The result of the present study together with previously published work on the biochemical PrP^d ^-typing of the more recent TSE isolates detected in 2004/2005 [24] indicate no evidence for BSE in the domestic small ruminant population in Switzerland. Nevertheless, the data derived from passive surveillance need careful interpretation, because they largely depend on disease awareness and compliance with the regulations. It is therefore difficult to assess how many suspicious sheep and goats had been missed or were not reported in the past. However, based on the results of the active surveillance that in principle overcomes these limitations, it appears highly unlikely that BSE became established in the small ruminant population in Switzerland.

Classical scrapie has never been considered an important threat in Switzerland. Indeed, the surveillance data imply that it is a very rare disease. Secondary cases were not found in the present study. Moreover, all scrapie cases were geographically separated and epidemiological follow-up investigations found no relation between the affected flocks (unpublished data). Disease transmission between and within flocks therefore appears very inefficient. A factor that may account for such a situation is a very low frequency of animals with classical scrapie susceptible PRNP genotypes. Unfortunately several attempts to extract genomic DNA from wax embedded tissues of the historic cases and their flock mates failed (unpublished data). However, for those of case S1/RS, DNA samples were available and more than half of them revealed a classical scrapie susceptible genotype. Moreover, in some of the affected sheep flocks (see additional file 2) and also in previous investigations it has been documented that such genotypes occur at rates ranging from approximately 30% to 60% in different domestic sheep breeds [33]. It can therefore be assumed that a proportion of the flock mates of the historic cases were indeed of susceptible genotypes. Environmental contamination is considered to substantially contribute to the persistence of classical scrapie in affected populations. There is evidence that the agent may remain infectious over years, if not decades, in the environment [38,39]. In Switzerland this factor does not appear to play an important role. This could be related to relatively small herd sizes and a low percentage of intensive farming in the Swiss small ruminant sector and differences in herd management and lambing practice compared to other countries [40].

It was rather unexpected, that only one case of classical scrapie has been identified in the 2004-2005 active surveillance program, which is considered much more efficient compared to passive surveillance alone. A possible explanation is that the prevalence of classical scrapie decreased compared to the mid 1990s when most of the other cases were identified. This could be the result of a very efficient passive surveillance and successful disease control or may be related to other yet unidentified factors that affect disease transmission. An interesting finding was that the two classical scrapie affected goats (IDs 15355 and 22614) originated from CAEV infected flocks. CAEV is closely related, if not identical, to maedi-visna virus in sheep; both belong to the genus lentivirus. There are several lines of evidence indicating that lentivirus infections play a role in the pathogenesis and transmission of scrapie [41-43]. CAE has been subject to mandatory eradication policies in Switzerland since 1994 and thereupon its prevalence has been drastically reduced. This situation may at least partially explain the absence of classical scrapie in goats in the 2004/2005 active surveillance sample. Although scientifically not proven, one could also speculate that the ban on MBM in ruminant feed since 1990 might have prevented recycling not only of BSE but also of classical scrapie to sheep and goats. Finally, we cannot exclude that single cases of classical scrapie eventually result from spontaneous PrP^c ^to PrP^d ^conversion events and occur as a sporadic TSE similar to sporadic TSEs in humans. However, at the time being such scenarios are highly speculative.

The number of atypical scrapie cases in the active surveillance was unexpectedly high. It can even be assumed that it has been underestimated to a certain extent in both passive and active TSE surveillance. Firstly, clinical signs of atypical scrapie apparently differ from those in classical scrapie [44,45]. Yet, if such cases were reported, histopathological changes and PrP^d ^depositions in the brainstem are absent or very mild and do not involve the target structures routinely investigated for the diagnosis of classical scrapie and BSE and consequently may have been missed (for review see [34]). Secondly, in the early years of active surveillance between 1998-2002 screening tests were applied that later on proved to fail in the detection of atypical scrapie cases [46]. Lastly, although the surveillance program 2004-2005 involved improved screening test formats, all slaughtered animals were analyzed on brainstem samples only, which is clearly less optimal compared to testing cerebellum samples or even more rostral brain structures for the detection of atypical scrapie cases. However, as mentioned before, the fallen stock is the most important target population, and for these animals other brain regions and multiple test formats were included in the diagnostic procedures in active surveillance of 2004-2005. Despite these limitations the prevalence of atypical scrapie in Switzerland was estimated comparable to most countries in the European Union [11]. The fact that only some secondary cases of atypical scrapie have been identified worldwide and that it has also been reported from countries with no or very few cases of classical scrapie, lead to the suggestion that the etiologies of classical scrapie and atypical scrapie are different and the transmission of atypical scrapie is less effective. This implies the possibility of a sporadic origin of atypical scrapie [47]. However, there are still major uncertainties related to the biodiversity of atypical scrapie isolates and the potential to cross over to other species still remains to be evaluated [48].

The Bayesian prevalence calculations proved what was suggested by observations before: the existence of cases of BSE is highly unlikely in the small ruminants' population and both atypical and classical scrapie is present on a very low level. Unfortunately, the one-year comprehensive survey did not yield the planned confidence to exclude cases of BSE, as due to logistical restrictions only 60% of the adult slaughtered animals could be tested.

Previously, estimation of the BSE prevalence in sheep has been linked to the apparent prevalence of classical scrapie [49]. In that study the estimate for the maximum proportion of scrapie cases that could be BSE was 0.02%, corresponding to 20 BSE-infected flocks in 2001 in Great Britain. Given the same relation of BSE/scrapie cases for Switzerland, the estimate would be 0.008 flocks (prevalence 0.041 BSE positive flocks per 100'000) with BSE for the 95% value of the calculated scrapie prevalence (41 cases assumed to be in 41 flocks). However, as we identified only 6 flocks with classical scrapie, applying the same calculation with our own data using the apparent prevalence would result in 0.0012 (prevalence 0.006 BSE positive flocks per 100'000) flocks with BSE. As there have been considerable doubts on the use of scrapie cases for the search of BSE in sheep [50], and the results calculated with this method were very low, we decided not to rely on this way of calculation for the BSE prevalence. Thus, given a more reliable method for prevalence calculation, our estimate of 0-15 BSE cases was only slightly below the estimated number of BSE cases in Great Britain in the year 2001. However, as this was yield with the method of BSE prevalence calculation from classical scrapie cases, our results can be seen as very conservative accordingly.

Models of small ruminant TSE have most often focused on predicting the human health risk from BSE in sheep [51,52] or deal mainly with the disease's dynamics on the flock level [53-57]. But on the national level one important question is, how likely TSEs will persist in the national flock [58,59]. With the simulation models presented in this paper it was possible to show that TSEs might persist over years in the population of small ruminants, which is in accordance with the surveillance results for classical scrapie. However, as parameters of variables were available for classical scrapie only, we used the same parameters for all three TSEs. But this approach is only feasible if transmission routes of the three TSEs are the same and transmission within each route is equally effective. The first point is still unanswered for animal-to-animal transmission, but in order to assess a worst-case scenario, our assumption was that the transmission routes are equal. There is no indication for both forms of scrapie of transmission via feed. For BSE, the transmission route via contaminated feed can be ruled out, as the feed ban in Switzerland is highly effective at least since the year 2000 [30]. For animal-to-animal transmission, all current evidence is in favor of a less efficient transmission of BSE and atypical scrapie compared to classical scrapie [11,60]. Thus, our parameters for BSE and atypical scrapie are worst-case assumptions, the real transmission risk being less than the risk we modeled. The parameters we used have been estimated from British data, and it can be questioned if this can be applied for the Swiss situation. Clearly, it would have been better to use Swiss data, but for most of the variables no such data was available. However, no great difference in the disease related parameters such as the incubation period can be expected. Additionally, there is no indication of differences in the genotype frequencies between GB and Switzerland [33]. For the infection probability of animals in uninfected flocks, we used an approach that is only driven by the actual disease situation in the model and the flock size. Thus, this important factor needs no parameterization. In the absence of sound data on movements of small ruminants between flocks, this simple approach offers an honest solution. Additionally, as in Switzerland a large proportion of small ruminants are kept in summer on common alpine pasture, using a method approximating homogenous mixing is plausible.

In the sensitivity analysis we showed that for R_0 _values below 5 there is a high probability for TSEs to fade-out of the Swiss small ruminant population within few decades. More recently published work shows a within-flock R_0 _for classical scrapie between 1.5 and 6 [56]. Indeed, our data from the investigations of flock mates suggest a very low R_0 _value and thus it seems that the R_0 _values in our original model were too high and the endemic persistence of classical scrapie, or even atypical scrapie and BSE in Switzerland is unlikely. However, if a spontaneous origin of atypical scrapie is the rule, then the disease will stay present at the observed level.

## Conclusions

In the wake of the BSE epidemic in cattle, fundamental concerns regarding a possible maintenance of the agent in the small ruminant population were raised. Based on the present study it is considered highly unlikely that the BSE agent was endemic in Swiss sheep and goats at the time when BSE in cattle faded out in 2006. However, statistically we cannot exclude that single cases occurred. In addition, the prevalence of classical scrapie was calculated as very low, but atypical scrapie was found at a higher rate. Even the forecast of the prevalence of small ruminant TSEs in a simulation model for the next decades in an unlikely worst-case scenario predicted that an epidemic of either type of small ruminant TSE in Switzerland cannot be expected. In view of the high costs related to active TSE surveillance in small ruminants the design of such programs must be subject of cost-benefit analysis. If this finally results in pure passive disease surveillance schemes, their efficiency must be provided by a high disease awareness and compliance.

## Authors' contributions

CH and AN performed the experiments and analyzed the data. CH and AO interpreted the immunohistochemistry. HS calculated the prevalences and simulation models. CH, DH, HS and TS wrote the manuscript. TS, DH, HS and AZ designed the study. All authors read and approved the final manuscript.

## Supplementary Material

Additional file 1 **Materials and Methods**. The data provide further details on the protocols for the PrP^d ^immunhistochemistry and the discriminatory Western immunoblot. Click here for file

Additional file 2 **Genetic analysis**. PRNP genotypes of flock mates of classical scrapie and atypical scrapie affected sheep and goats are presented. Click here for file
