# Supplementary material for: Surveillance and simulation of bovine spongiform encephalopathy and scrapie in small ruminants in Switzerland
Source: BMC Vet Res. 2010 Apr 18;6:20. doi: 10.1186/1746-6148-6-20 (PMC2867968; doi:10.1186/1746-6148-6-20)
Supplement: Additional file 1 — Materials and Methods. The data provide further details on the protocols for the PrPd immunhistochemistry and the discriminatory Western immunoblot. [file 1746-6148-6-20-S1.DOC]

**Material and Methods**

**Immunohistochemistry (IHC)**

Four µm-thick tissue sections were cut from the paraffin blocks, floated on adhesive treated slides (Superfrost® PLUS, Menzel GmbH &co, KG) and dried overnight at 37°C. Next, the slides were deparaffinised and rehydrated in 3 changes of xylene followed by 3 changes in increasing concentrations of ethanol (70%, 80% and 95%) for 2 min each. Two treatments are consecutively used for antigen retrieval: immersion in undiluted formic acid (98%) for 30 min followed by 10 min rinsing in tap water and hydrated autoclaving (45 min, 121°C, 1 bar) in citrate buffer, pH 6 (target retrieval solution, Dako). After cooling down, the endogenic peroxidase was inhibited by incubation in 3% (v/v) hydrogen peroxide in absolute methanol for 10 min.

Then the slides were rinsed with TBS and blocked in 5% normal goat serum for 20 min. Depending on the purpose of the IHC analysis different PrP specific monoclonal antibodies (MAb) were used. Irrespective of the antibody applied, the slides were incubated for one hour at room temperature and rinsed three times with TBS. The following detection steps were performed by using two different commercial kits according to the instructions of the manufacturers: Vectastain® Elite® ABC Kit for mAb R145 and Dako REAL^TM^ EnVision^TM^ Detection System K5007 for all other mAbs respectively. Counterstaining was carried out with haematoxyline.

**Discriminatory Western Blot (WB)**

In principle the discriminatory WB was carried out on the basis of a commercial BSE rapid test (Prionics Check Western, Prionics Switzerland) and as described previously by M. Stack and collegues [27]. Briefly, tissue homogenates were digested with Proteinase K for 40 min at 48° C and boiled in SDS PAGE sample buffer. The samples were loaded in parallel and in identical order on two pre-cast ten-well 12% NuPage Gels (Invitrogen), separated by SDS-PAGE at 150 V for 90 min and tank-blotted to PVDF membranes (Milipore) for 60 min at 150V. The membranes were blocked and incubated separately with two different mAbs, namely the PrP core-binding 6H4 (0,2 µg/ml, Prionics) and the N-terminal-binding mAb P4 (0,1 µg/ml, r-biopharm). After detection by an alkaline-phosphatase conjugated secondary antibody and CDP-Star substrate (Roche), the membranes were exposed to a photographic film and developed using standard procedures.
