# Supplementary material for: Surveillance and simulation of bovine spongiform encephalopathy and scrapie in small ruminants in Switzerland
Source: BMC Vet Res. 2010 Apr 18;6:20. doi: 10.1186/1746-6148-6-20 (PMC2867968; doi:10.1186/1746-6148-6-20)
Supplement: Additional file 2 — Genetic analysis. PRNP genotypes of flock mates of classical scrapie and atypical scrapie affected sheep and goats are presented. [file 1746-6148-6-20-S2.DOC]

Genotypes of flock mates of classical and atypical scrapie cases identified in the active surveillance program, 2004-2005.

| Scrapie type | Case ID | *PRNP* polymorphisms 136, 154, 171 | | | | | | | |
| --- | --- | --- | --- | --- | --- | --- | --- | --- | --- |
|  |  | VRQ/VRQ | ARQ/VRQ | ARR/VRQ | ARQ/ARQ | AHQ/ARQ | AHQ/AHQ | ARR/ARQ | ARR/ARR |
| Atypical Scrapie | S7/CS (sheep) | 1 | 9 |  | 16 |  |  | 2 |  |
|  | G2/FS (goat) |  |  |  |  | 1 |  |  |  |
|  | G1/RS (goat) |  |  |  | 13 | 4 |  |  |  |
|  | S5/FS (sheep) |  | 3 | 7 | 2 |  |  | 12 | 5 |
|  | S6/FS (sheep) | 1 | 1 | 2 |  |  |  | 5 | 7 |
| Classical scrapie | S1/RS (sheep) | 2 | 10 | 6 | 10 |  |  | 20 | 8 |
